# First report and complete genome analysis of infectious bronchitis virus from retailed chicken meat in Mongolia in 2023

**DOI:** 10.3389/fvets.2024.1465342

**Published:** 2024-12-13

**Authors:** Sun-Hak Lee, Heesu Lee, Andrew Y. Cho, Tae-Hyeon Kim, Yun-Jeong Choi, Nyamsuren Otgontogtokh, Ye-Ram Seo, Dong-Yeop Lee, Erdene-Ochir Tseren-Ochir, Temuulen Myagmarsuren, Dong-Hun Lee, Ji-Yeon Hyeon, Chang-Seon Song

**Affiliations:** ^1^Avian Disease Laboratory, College of Veterinary Medicine, Konkuk University, Seoul, Republic of Korea; ^2^Wildlife Health Laboratory, College of Veterinary Medicine, Konkuk University, Seoul, Republic of Korea; ^3^Department of Infectious Diseases and Microbiology, School of Veterinary Medicine, Mongolian University of Life Sciences, Ulaanbaatar, Mongolia

**Keywords:** infectious bronchitis virus (IBV), retailed chicken meat, grocery market, whole genome sequencing, recombination

## 1 Introduction

Infectious bronchitis virus (IBV) causes considerable economic losses in poultry industry due to respiratory diseases, decreased egg production, and increased mortality in chickens (1). The virus, a member of the genus *Gammacoronaviruse*s and family *Coronaviridae*, has a highly mutable RNA genome that facilitates the rapid emergence of new variants (2). Its genome consists of single-stranded positive-sense RNA (~27.6 kb in size), which encodes four structural proteins [spike (S), envelope (E), membrane (M), and nucleocapsid (N)] (3) and several non-structural proteins, including two polyproteins (pp1a and pp1ab), and accessory proteins (3a, 3b, 5a, and 5b) (4, 5).

The IBV strains were categorized into six genotypes encompassing 32 distinct viral lineages based on the full S1 gene sequence (6). The geographic distribution and diversity of IBV lineages varied significantly. Some lineages, such as the Mass-type, 4/91 (793 B or CR88)-like, D274-like, D3128, QX-like, and Italy02, are globally widespread (7). Conversely, the GI-23 lineage, which includes Variant-2 viruses, is predominantly found in the Middle East (6).

Despite the distinct origins, evolution, and transmission dynamics of each genotype, several common factors including live poultry trade and use of exogenous live vaccines contribute to their widespread distribution across various countries (8). For example, the GI-5 and GI-6 genotypes of IBV, originating from the Australian vaccine strains JAAS and J9, respectively, introduced into China, have contributed to the genetic diversity observed in IBV populations (9).

The global spread of IBV underscores the critical need for comprehensive surveillance. Early detection through active surveillance of poultry flocks or products can identify novel IBVs before it becomes widespread, aiding in virus control and contributing to global efforts to track its evolution and transmission dynamics (10).

In this study, we detected a novel IBV strain in retail chicken meat, sampled in a retail grocery market in Mongolia in 2023. For whole-genome sequencing of the IBV isolate, we utilized the Sequence-Independent Single-Primer Amplification (SISPA) protocol for next-generation sequencing. We conducted a comprehensive phylogenetic analysis of the S1 gene to determine the genetic relatedness of the isolated IBVs to the globally distributed IBVs. Additionally, comparative genome analysis revealed recombination events in the IBV isolate, highlighting its genetic diversity and evolutionary complexity.

## 2 Materials and methods

### 2.1 Sample collection and virus isolation

On August 21, 2023, six chicken meat samples were purchased from a retail grocery market in Ulaanbaatar, Mongolia. Four of these chickens were locally sourced from Mongolia, whereas the remaining two were imported from Russia, processed in April 2023, and distributed in a frozen state. Each chicken meat sample was placed separately in a sterile plastic bag containing 400 mL of phosphate buffered saline (PBS) and shaken for 2 min. The resulting rinse was clarified using centrifugation at 3,000 rpm for 10 min and filtered through a sterile 0.45 μm Minisart^®^ Syringe Filter (Sartorius, Göttingen, Germany). Viruses were isolated via the allantoic route in 10-day-old specific-pathogen-free (SPF) chicken embryos, with each embryo receiving a 0.2 mL inoculation of the supernatant from the rinse solution, followed by incubation at 37°C for 72 h. The allantoic fluid harvested from the eggs was tested for hemagglutination (HA) assay to screen viruses including avian influenza virus (11). RNA was extracted from the allantoic fluid using a Qiagen RNeasy Mini Kit (Hilden, Germany) and IBV was detected using real-time quantitative reverse transcription PCR (qRT-PCR) as previously described (12), using Qiagen Quantitect RT-PCR reagents (Qiagen, Manchester, UK).

### 2.2 NGS preparation using SISPA

The SISPA method was employed for amplification of the RNA viral genomes utilizing random priming, as previously described (13). Briefly, cDNA was synthesized from DNase I-treated RNA using the SuperScript™ IV First-Strand Synthesis System (Thermo Fisher Scientific, Waltham, MA, USA) with primer K-8N. The complementary strand of cDNA was synthesized using DNA Polymerase I, Large (Klenow) Fragment [New England Biolabs (NEB), Ipswich, MA, USA], and the primer K-8 N, resulting in dsDNA. The resulting products were purified using a QIAquick PCR Purification Kit (QIAGEN, Hilden, Germany). Purified dsDNA was amplified using a Phusion High-Fidelity PCR kit (New England Biolabs, Ipswich USA, MA) with primer K, followed by purification using the same method. All procedures were performed according to manufacturer's instructions. Library preparation and sequencing were performed at Bionics (Seoul, Republic of Korea) using an Illumina MiSeq next-generation sequencing (NGS) system (San Diego, California, USA).

### 2.3. Assembly and phylogenetic analysis

The resulting raw reads were trimmed to remove adapters and low-quality bases using BBDuk v35.49. After *de novo* assembly of the trimmed reads using Geneious assembler, reference mapping was performed using Geneious mapper in Geneious G9.0.5(Biomatters, Auckland, New Zealand). A reference sequence (GenBank accession no. KJ135013) was selected based on BLAST results of contigs.

To determine the genotype and lineage based on the S1 phylogeny-based classification system (6), S1 gene sequences, including representative sequences for each genotype, were obtained from GenBank (Supplementary Table 1). To investigate the genetic relationship with global IBV lineages, the S1 gene sequences of the determined lineage and sequences with high nucleotide sequence identities (based on BLASTn results) were downloaded from the GenBank database (Supplementary Table 2). All sequences were aligned using MAFFT v7.308 for multiple sequence alignment (14). A maximum-likelihood phylogenetic tree was constructed using RAxML with a general time-reversible (GTR) nucleotide model and gamma distribution, along with 1,000 rapid bootstrap replicates (15).

### 2.4 Recombination analysis

Potential recombination events were investigated using Recombination Detection Program (RDP) 5 software (v.5.56) employing multiple detection methods, including RDP, GENECONV, BootScan, MaxChi, Chimera, SiScan, Phylpro, LARD, and 3Seq, as previously described (16). Recombination events were considered significant when at least five detection methods yielded a *p*-value < 0.01. A confirmatory analysis of potential recombination events and breakpoints was conducted using the SimPlot Program (version 3.5.1) and BootScan analyses (17). Nucleotide identity comparisons were performed using the Kimura 2-parameter method with a transition-transversion ratio of 2, a window size of 500 bp, and a step size of 50 bp. BootScan analyses were conducted using the neighbor-joining method with 100 replicates to ensure robust validation of recombination events. Recombination breakpoints were analyzed by maximization of χ^2^ using the program Findsites included in the SimPlot. The similarity plot displays the percentage of permuted trees (%) among the queried strain and parental strains. Strains were considered as recombinants if any crossover event took place between three putative parental strains.

## 3 Descriptive results

Among the six samples, we isolated the MR23/6/Mongolia/2023 (MR23/6) virus from a chicken sample imported from Russia using chicken embryo inoculation and confirmed the presence of IBV by real-time qRT-PCR, while none of the samples showed positive for HA activity in the HA assay. A total of 211,210 NGS reads were produced, resulting in a contig length of 27,526 bp and 14 coding sequences (CDSs). The complete genome was deposited in the GenBank database under accession no. PP871397.

**Figure 1:**
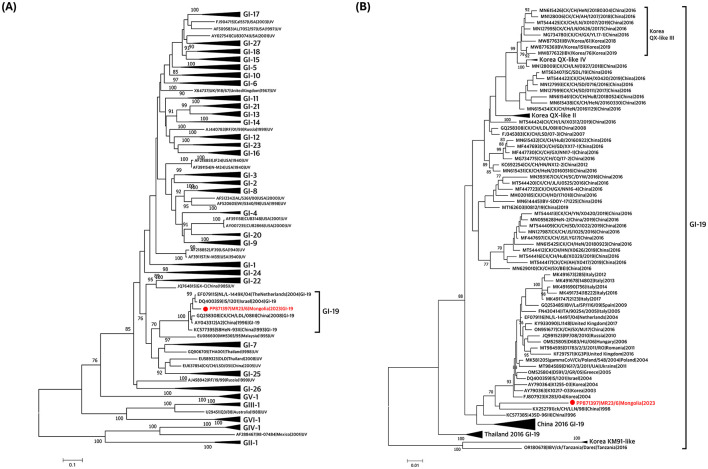
Phylogenetic trees based on alignment of S1 gene sequences of the Mongolian infectious bronchitis virus (IBV) isolate (red) and reference strains. The trees were created by the maximum-likelihood method in RAxML using Tamura-Nei model and 1,000 bootstrap replicates. The IBVs newly isolated in this study are shown in red with a circle. **(A)** S1 gene-based phylogenetic tree was constructed using 191 reference sequences. All lineages except for GI-19 lineage were compressed. **(B)** S1 gene-based phylogenetic tree was constructed using 101 IBV strains previously reported as GI-19 lineage. Korean GI-19 genotypes, Chinese GI-19 lineage in 2016, and Thailand GI-19 lineage in 2016 were compressed.

Classification based on the S1 gene confirmed that the virus belonged to the GI-19 lineage (Figure 1A, Supplementary Figure 1A). Phylogenetic analysis of GI-19 lineage viruses revealed that the isolate clustered with other GI-19 lineage viruses from Asia (Korea and China) and Europe (Poland, Ukraine, the United Kingdom, the Netherlands, Italy, and Spain; Figure 1B, Supplementary Figure 1B). It formed a distinct long branch, indicating that no highly similar viruses have been identified, suggesting the need for additional genomic surveillance.

**Figure 2:**
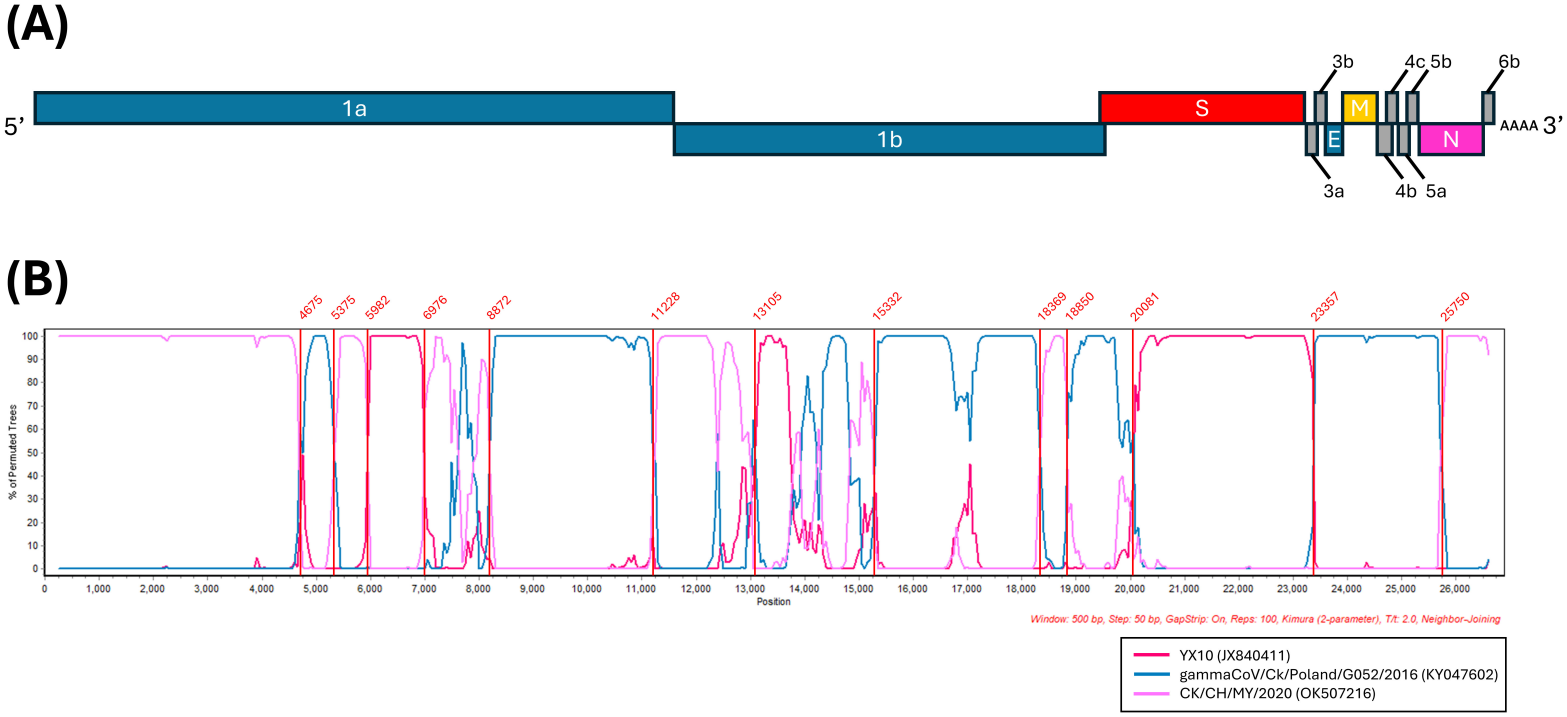
Multiple recombination events detected in the Mongolian IBV isolate. **(A)** Genome organization of the Mongolian IBV isolate (MR23/6). **(B)** Simplot analysis was performed to detect recombination in the whole genome from two Chinese IBV isolates, CK/CH/MY/2020 (JX840411) and YX10 (JX840411), and one Poland IBV isolate, gammaCoV/Ck/Poland/G052/2016 (KY047602). Recombination breakpoints analyzed by maximization of χ^2^ using the program Findsites included in the SimPlot were indicated within the plot (lines and numbers in red). Bootscan evidence for the recombination origin analyzed with Simplot based on pairwise distance, modeled with a window size of 500 bp and a step size of 50 bp, and 100 Bootstrap replicates.

BLAST search of the complete genome revealed the highest similarity to the CK/CH/MY/2020 strain identified in China, with a sequence identity of 92.66%. BLAST search of each CDS identified genetically similar IBVs in a range of 90.07%−100% sequence identity (Supplementary Table 3). Based on these results, 22 reference strains were selected for the analysis of potential recombination events. The RDP analysis confirmed five recombination events (Supplementary Table 4). The IBV strains YX10, CK/CH/MY/2020, and gammaCoV/Ck/Poland/G052/2016, derived from the RDP analysis, were used as putative parental strains for SimPlot analysis when the MR23/6 was queried (Figure 2). The similarity plot also identified three putative parental strains, the YX10 and CK/CH/MY/2020 originated from China, belonging to the GI-19 and GI-28 lineage, respectively (18, 19) and the gammaCoV/Ck/Poland/G052/2016 belonging to the GI-23 (Var2-like) lineage, which traces its origin back to the Middle East (20). Reassortant IBVs produced by recombination between IBVs of Chinese and European origin also have been reported in Italy (21, 22) and Spain (22), highlighting frequent reassortment of IBVs in Eurasia.

In conclusion, we report the first isolation and genome sequences of reassortant GI-19 IBV identified from retail chicken meat in Mongolia. Our findings also highlight the practicality of utilizing retail chicken meat samples from grocery markets for pathogen surveillance, circumventing the necessity for direct sampling of live animals.

## Data Availability

The datasets presented in this study can be found in online repositories. The names of the repository/repositories and accession number(s) can be found in the article/Supplementary material.

## Supplementary material

Supplementary Table 1 Metadata for infectious bronchitis virus sequences (*n* = 190) used in S1 gene based phylogenetic analysis (Supplementary Figure 1A).

Supplementary Table 2 Metadata for GI-19 lineage infectious bronchitis virus sequences (*n* = 102) used in S1 gene based phylogenetic analysis (Supplementary Figure 1B).

Supplementary Table 3 Pairwise comparison of nucleotide homology of coding sequences (CDS) between the Mongolian infectious bronchitis virus (MR23-6) and other IBV strains.

Supplementary Table 4 Recombination events detected in whole genome of the Mongolian infectious bronchitis virus isolate (MR23/6).

Supplementary Figure 1 **(A)** Phylogenetic trees based on alignment of S1 gene sequences of the Mongolian infectious bronchitis virus (IBV) isolate (red) and reference strains. The trees were created by the maximum-likelihood method in RAxML using Tamura-Nei model and 1,000 bootstrap replicates. The IBVs newly isolated in this study are shown in red with a circle. S1 gene-based phylogenetic tree was constructed using 191 reference sequences. **(B)** Phylogenetic trees based on alignment of S1 gene sequences of the Mongolian infectious bronchitis virus (IBV) isolate (red) and reference strains. The trees were created by the maximum-likelihood method in RAxML using Tamura-Nei model and 1,000 bootstrap replicates. The IBVs newly isolated in this study are shown in red with a circle. S1 gene-based phylogenetic tree was constructed using 101 IBV strains previously reported as GI-19 lineage.
