# Supplementary material for: First report and complete genome analysis of infectious bronchitis virus from retailed chicken meat in Mongolia in 2023
Source: Front Vet Sci. 2024 Dec 13;11:1465342. doi: 10.3389/fvets.2024.1465342 (PMC11671396; doi:10.3389/fvets.2024.1465342)
Supplement: Supplementary Table 1 — Metadata for infectious bronchitis virus sequences (n = 190) used in S1 gene based phylogenetic analysis (Supplementary Figure 1A). [file Table_1.docx]

Supplementary Table 1. Metadata for infectious bronchitis virus sequences (n=190) used in S1 gene based phylogenetic analysis (Figure 1 (A), Supplementary Figure 1).

| Accession No. | ID | lineage | year | lineage | Accession No. | ID | lineage | year | lineage |
| --- | --- | --- | --- | --- | --- | --- | --- | --- | --- |
| EF079115 | NL/L-1449K/04 | The Netherlands | 2004 | GI-19 | KC577388 | 48SD-96VI | China | 1996 | GI-18 |
| DQ400359 | IS/1201 | Israel | 2004 | GI-19 | FJ807932 | B4 | Korea | 1986 | GI-15 |
| AY043312 | A2 | China | 1996 | GI-19 | FJ807944 | K620/02 | Korea | 2002 | GI-15 |
| GQ258308 | CK/CH/LDL/08III | China | 2008 | GI-19 | FJ807933 | EJ95 | Korea | 1995 | GI-15 |
| KC577395 | 58HeN-93II | China | 1993 | GI-19 | JQ977697 | SNU8067 | Korea | 2008 | GI-15 |
| EU086600 | MH5365/95 | Malaysia | 1995 | UV | AY257068 | K210-02 | Korea | 2002 | GI-15 |
| GQ906705 | THA001 | Thailand | 1998 | UV | AY257062 | K281-01 | Korea | 2001 | GI-15 |
| KC478591 | SC1203 | China | 2012 | GI-7 | X64737 | UK/918/67 | United Kingdom | 1967 | UV |
| GQ229232 | 3382 | Taiwan | 2006 | GI-7 | KF809791 | IBV422 | India | 2012 | GI-24 |
| JQ764816 | GX-G | China | 1988 | GI-7 | KF809795 | IBV470 | India | 2013 | GI-24 |
| JQ739299 | ck/CH/LHLJ/110664 | China | 2011 | GI-7 | KF809796 | IBV506 | India | 2013 | GI-24 |
| AY606322 | T07/02 | Taiwan | 2002 | GI-7 | KF757451 | V25 | India | 2004 | GI-24 |
| AY606320 | TP/64 | Taiwan | 1964 | GI-7 | KF757450 | V24 | India | 2007 | GI-24 |
| KM660636 | GA/10216/2010 | USA | 2010 | GI-25 | KF757447 | V13 | India | 1998 | GI-24 |
| EU925393 | CA/1737/04 | USA | 2004 | GI-25 | AF391157 | N-M39 | USA | 1940 | UV |
| EU694402 | DMV/5642/06 | USA | 2006 | GI-25 | AF218852 | JF39 | USA | 1940 | UV |
| KP085595 | GA/12274/2012 | USA | 2012 | GI-25 | KF411040 | CK/CH/LLN/111169 | China | 2011 | GI-1 |
| JN160805 | GA/60173/2007 | USA | 2007 | GI-25 | KF809793 | IBV438 | India | 2012 | GI-1 |
| KP085597 | GA/13485/2013 | USA | 2013 | GI-25 | KJ200289 | ckZA/6743b/11 | South Africa | 2011 | GI-1 |
| EU589323 | DLD | Thailand | 2008 | UV | FJ888351 | H120 | The Netherlands | 1960 | GI-1 |
| EU637854 | CK/CH/LSD/05I | China | 2005 | UV | M95169 | Beaudette | USA | 1937 | GI-1 |
| JQ764815 | GX-C | China | 1985 | UV | AY561711 | M41 | USA | 1941 | GI-1 |
| DQ288927 | SAIBK | China | 2007 | GI-22 | JX182787 | UFMG/283 | Brazil | 1983 | GI-11 |
| DQ167147 | CK/CH/LSC/99I | China | 1999 | GI-22 | JX182785 | UFMG/297 | Brazil | 1983 | GI-11 |
| GQ265940 | HN08 | China | 2008 | GI-22 | JX182775 | UFMG/G | Brazil | 1975 | GI-11 |
| KC577397 | 66GD-98VI | China | 1998 | GI-22 | GU393339 | IBV/Brasil/351/1984 | Brazil | 1984 | GI-11 |
| HQ018890 | CK/CH/JX/JA09-1 | China | 2009 | GI-22 | JX182783 | UFMG/1141 | Brazil | 2009 | GI-11 |
| KC577382 | 40GDGZ-97I | China | 1997 | GI-22 | JX182777 | UFMG/12.2 | Brazil | 2008 | GI-11 |
| AY007235 | CU82866 | USA | 2000 | UV | AJ441314 | RF/01/02 | Russia | 2002 | GI-12 |
| AF391158 | CU83148 | USA | 2001 | UV | X52084 | D3896 | The Netherlands | 1978 | GI-12 |
| AF349621 | Qu_mv | Canada | 1996 | GI-20 | M21969 | D207 | The Netherlands | 1979 | GI-12 |
| AF349620 | Qu16 | Canada | 1996 | GI-20 | FN182276 | NGA/295/2006 | Nigeria | 2006 | GI-12 |
| GQ844988 | Guangzhou-06 | China | 2006 | GI-2 | X15832 | D274 | The Netherlands | 1979 | GI-12 |
| DQ070840 | SDW | China | 2004 | GI-2 | X04723 | 6/82 | United Kingdom | 1982 | GI-12 |
| GU393336 | Holte | USA | 1954 | GI-2 | AY091552 | Israel/720/99 | Israel | 1999 | GI-23 |
| GU393337 | Iowa97 | USA | 1956 | GI-2 | AF093796 | Variant2 | Israel | 1998 | GI-23 |
| GU361608 | Iowa609 | USA | 1956 | GI-2 | KC533682 | Eg/1265B/2012 | Egypt | 2012 | GI-23 |
| JQ964061 | L165 | USA | 1965 | GI-8 | EU780077 | IS/1494/06 | Israel | 2006 | GI-23 |
| M99484 | SE17 | USA | 1967 | GI-8 | KJ941019 | IZO28/86 | Italy | 1986 | GI-16 |
| JQ964071 | L919 | USA | 1966 | GI-8 | AY606323 | 2992 | Taiwan | 2002 | GI-16 |
| JQ964067 | L718 | USA | 1966 | GI-8 | EF030995 | CK/CH/LDL/97I | China | 1997 | GI-16 |
| JQ964070 | L905 | USA | 1966 | GI-8 | AF227438 | T3 | China | 1996 | GI-16 |
| JQ964066 | L613 | USA | 1965 | GI-8 | AF286302 | Q1 | China | 1996 | GI-16 |
| AF352831 | BL-56 | Mexico | 1996 | GI-3 | GU938413 | CK/CH/Chongqing/0908 | China | 2009 | GI-16 |
| GQ229245 | 3381 | Taiwan | 2006 | GI-3 | AJ440783 | RF/01/99 | Russia | 1999 | UV |
| L14070 | JMK | USA | 1964 | GI-3 | FN182277 | NGA/324/2006 | Nigeria | 2006 | GI-14 |
| L14069 | Gray | USA | 1960 | GI-3 | X87238 | B1648 | Belgium | 1984 | GI-14 |
| AY789947 | PA/5344/98 | USA | 1998 | GI-3 | DQ386105 | Spain/04/5438 | Spain | 2004 | GI-21 |
| AF520606 | IA/10623/99 | USA | 1999 | GI-3 | DQ064808 | Spain/98/313 | Spain | 1998 | GI-21 |
| AF520605 | WI/5340/98 | USA | 1998 | UV | DQ064806 | Spain/97/314 | Spain | 1997 | GI-21 |
| AF512342 | AL/5361/00 | USA | 2000 | UV | AJ457137 | Italy02 | Italy | 1999 | GI-21 |
| AF391154 | N-M24 | USA | 1940 | UV | DQ901377 | It/497/02 | Italy | 2002 | GI-21 |
| AF218851 | JF24 | USA | 1940 | UV | DQ901376 | UK/L-633/04 | United Kingdom | 2004 | GI-21 |
| DQ912831 | CAL99 | USA | 1999 | GI-9 | DQ386098 | Spain/00/336 | Spain | 2000 | GI-13 |
| AF519573 | IA/3589/98 | USA | 1998 | GI-9 | EU914938 | Moroccan-G/83 | Morocco | 1983 | GI-13 |
| M99482 | ARK99 | USA | 1973 | GI-9 | AF093795 | Variant1 | Israel | 1996 | GI-13 |
| JQ739363 | ck/CH/LSD/110712 | China | 2011 | GI-9 | AJ618985 | FR-85131-85 | France | 1985 | GI-13 |
| KC577392 | 54HaN-95I | China | 1995 | GI-9 | Z83975 | UK/7/91 | United Kingdom | 1991 | GI-13 |
| AF006624 | ArkDPI | USA | 1981 | GI-9 | JQ739375 | ck/CH/LSD/110857 | China | 2011 | GI-13 |
| U29453 | N3/62 | Australia | 1962 | GI-5 | L18988 | Holte | USA | 1962 | GI-4 |
| U29522 | N1/62 | Australia | 1962 | GI-5 | KC577394 | 56GX-98I | China | 1998 | GI-4 |
| AY839140 | JAAS-vaccine strain | China | 2004 | GI-5 | AY251816 | GX2-98 | China | 2003 | GI-4 |
| AY775551 | HN99 | China | 1999 | GI-5 | FN182270 | NGA/N545/2006 | Nigeria | 2006 | GI-26 |
| DQ490215 | V2-02 | Australia | 2002 | GI-5 | FN182269 | NGA/N544/2006 | Nigeria | 2006 | GI-26 |
| DQ490205 | Armidale-vaccine strain | Australia | 2004 | GI-5 | FN182272 | NER/28/2007 | Niger | 2007 | GI-26 |
| U29523 | N2/75 | Australia | 1975 | GI-6 | FN182266 | NGA/BB91/2007 | Nigeria | 2007 | GI-26 |
| U29519 | VicS | Australia | 1960 | GI-6 | FN182268 | NGA/BP61/2007 | Nigeria | 2007 | GI-26 |
| AF151953 | A | New Zealand | 1970 | GI-6 | FN182243 | NGA/B401/2006 | Nigeria | 2006 | GI-26 |
| JQ250818 | QS | China | 2010 | GI-6 | AJ458942 | RF/19/99 | Russia | 1999 | UV |
| DQ515802 | J9-vaccinestrain | China | 2006 | GI-6 | FJ235191 | V1/07 | Australia | 2007 | GV-1 |
| U29520 | V5/90 | Australia | 1990 | GI-6 | JX018208 | 18 | Australia | 2008 | GV-1 |
| AF151954 | B | New Zealand | 1970 | GI-10 | FJ235194 | N1/03 | Australia | 2003 | GV-1 |
| AF151960 | T6 | New Zealand | 2000 | GI-10 | DQ059618 | N4/02 | Australia | 2002 | GV-1 |
| AF151956 | D | New Zealand | 1970 | GI-10 | DQ059620 | N4/03 | Australia | 2003 | GV-1 |
| AF151955 | C | New Zealand | 1970 | GI-10 | DQ059619 | N5/03 | Australia | 2003 | GV-1 |
| AF151959 | K87 | New Zealand | 2000 | GI-10 | U29451 | Q3/88 | Australia | 1988 | UV |
| AF151958 | K43 | New Zealand | 2000 | GI-10 | JN176213 | N1/08 | Australia | 2008 | GIII-1 |
| AY027541 | CU83074 | USA | 2001 | UV | U29450 | N1/88 | Australia | 1988 | GIII-1 |
| KM660635 | GA/13384/2013 | USA | 2013 | GI-27 | U29521 | V18/91 | Australia | 1991 | GIII-1 |
| KM660634 | GA/12341/2012 | USA | 2012 | GI-27 | DQ490219 | V6-92 | Australia | 1992 | GIII-1 |
| KM660631 | GA/10231/2010 | USA | 2010 | GI-27 | JF804680 | K119/09 | Korea | 2009 | GVI-1 |
| GU437864 | GPL8264 | USA | 2009 | GI-27 | KF007209 | SDIB781/2012 | China | 2012 | GVI-1 |
| GU437858 | GPL8225 | USA | 2008 | GI-27 | JX292013 | GX-NN09032 | China | 2009 | GVI-1 |
| GU301925 | GA08 | USA | 2008 | GI-27 | JF804677 | K23/10 | Korea | 2010 | GVI-1 |
| AF509583 | AL/7052/97 | USA | 1997 | UV | GQ265948 | TC07-2 | China | 2007 | GVI-1 |
| FJ904715 | Cal557 | USA | 2003 | UV | JF804687 | K273/09 | Korea | 2009 | GVI-1 |
| FJ904714 | Cal1995 | USA | 1995 | GI-17 | M21971 | D1466 | The Netherlands | 1979 | GII-1 |
| AF419315 | CA/Machado/88 | USA | 1988 | GI-17 | M21968 | V1397 | The Netherlands | 1984 | GII-1 |
| AF027509 | CV-56b | USA | 1991 | GI-17 | AF288467 | 98-07484 | Mexico | 2001 | UV |
| AF419314 | PA/171/99 | USA | 1999 | GI-17 | AF317212 | CU82616 | USA | 2000 | GIV-1 |
| AF510656 | AL/6609/98 | USA | 1998 | GI-17 | AF274436 | AR/6386/97 | USA | 1997 | GIV-1 |
| AY296745 | JP8443 | Japan | 1994 | GI-18 | EU283066 | GA/Avial1/vaccine strain | USA | 1991 | GIV-1 |
| AY296746 | JP9758 | Japan | 1995 | GI-18 | AF338719 | GA/13055/00 | USA | 2000 | GIV-1 |
| AY296744 | JP8127 | Japan | 1993 | GI-18 | AF274439 | GA/5381/99 | USA | 1999 | GIV-1 |
| KC577391 | 53XJ-99II | China | 1999 | GI-18 | U77298 | DE/072/92 | USA | 1992 | GIV-1 |

Supplementary Table 2. Metadata for GI-19 lineage infectious bronchitis virus sequences (n=102) used in S1 gene based phylogenetic analysis (Figure 1 (B), Supplementary Figure2).

| Accession No. | ID | country | year | lineage | Accession No. | ID | country | year | lineage |
| --- | --- | --- | --- | --- | --- | --- | --- | --- | --- |
| OR180678 | IBV/ck/Tanzania/Dares | Tanzania | 2016 | GI-19 | MT544409 | CK/CH/SD/X1022/2019 | China | 2016 | GI-19 |
| MW877665 | IBV/Korea/181 | Korea | 2020 | GI-19 | MT544413 | CK/CH/YN/X0420/2019 | China | 2016 | GI-19 |
| MG191027 | TH/IBV/2016/CU-109 | Thailand | 2016 | GI-19 | MT544412 | CK/CH/HN/X0626/2019 | China | 2016 | GI-19 |
| MG191022 | TH/IBV/2016/CU-104 | Thailand | 2016 | GI-19 | MT544417 | CK/CH/AH/X0417/2019 | China | 2016 | GI-19 |
| MG191038 | TH/IBV/2016/CU-120 | Thailand | 2016 | GI-19 | MN393167 | CK/CH/SC/DYW/2016 | China | 2016 | GI-19 |
| MG191014 | TH/IBV/2016/CU-93 | Thailand | 2016 | GI-19 | MW877644 | IBV/Korea/17 | Korea | 2018 | GI-19 |
| MG191040 | TH/IBV/2016/CU-124 | Thailand | 2016 | GI-19 | MW877645 | IBV/Korea/63 | Korea | 2016 | GI-19 |
| MG191024 | TH/IBV/2016/CU-106 | Thailand | 2016 | GI-19 | MW877654 | IBV/Korea/166 | Korea | 2016 | GI-19 |
| MG191037 | TH/IBV/2016/CU-119 | Thailand | 2016 | GI-19 | MW877646 | IBV/Korea/37 | Korea | 2017 | GI-19 |
| MG191011 | TH/IBV/2016/CU-90 | Thailand | 2016 | GI-19 | MW877649 | IBV/Korea/33 | Korea | 2017 | GI-19 |
| MG191025 | TH/IBV/2016/CU-107 | Thailand | 2016 | GI-19 | MN128014 | CK/CH/SX/0705/2018 | China | 2016 | GI-19 |
| MN615431 | CK/CH/HeN/20160516 | China | 2016 | GI-19 | ON260866 | CK/CH/GD/KPLH_CZQ/2018 | China | 2016 | GI-19 |
| KC692254 | CK/CH/HN/NX12-2 | China | 2012 | GI-19 | MN615448 | CK/CH/HuN/20160909 | China | 2016 | GI-19 |
| GQ258308 | CK/CH/LDL/08III | China | 2008 | GI-19 | MF447710 | CK/CH/GD/XX17-3 | China | 2016 | GI-19 |
| FJ345383 | CK/CH/LSD/07-3 | China | 2007 | GI-19 | MT544419 | CK/CH/YN/X1210/2019 | China | 2016 | GI-19 |
| MG734775 | CK/CH/CQ/17-2 | China | 2016 | GI-19 | MF447688 | CK/CH/GD/XX16-12 | China | 2016 | GI-19 |
| MF447730 | CK/CH/GX/NN17-1 | China | 2016 | GI-19 | MN393171 | CK/CH/SC/MS/2017 | China | 2016 | GI-19 |
| MN615432 | CK/CH/HuB/20160922 | China | 2016 | GI-19 | MT544418 | CK/CH/GX/X0115/2019 | China | 2016 | GI-19 |
| MF447693 | CK/CH/GD/XX17-1 | China | 2016 | GI-19 | MF447711 | CK/CH/GD/XX17-4 | China | 2016 | GI-19 |
| MT544424 | CK/CH/LN/X0312/2019 | China | 2016 | GI-19 | MF447720 | CK/CH/GX/NN17-3 | China | 2016 | GI-19 |
| MN615434 | CK/CH/HeN/20161129 | China | 2016 | GI-19 | MN615435 | CK/CH/HuB/20160901 | China | 2016 | GI-19 |
| MN127999 | CK/CH/SD/0111/2017 | China | 2016 | GI-19 | MN615439 | CK/CH/HeN/20161009 | China | 2016 | GI-19 |
| MW877666 | IBV/Korea/264 | Korea | 2019 | GI-19 | MN615440 | CK/CH/HuB/20160928 | China | 2016 | GI-19 |
| MT563407 | SC/SDL/19 | China | 2016 | GI-19 | MG734787 | CK/CH/GX/NN17-6 | China | 2016 | GI-19 |
| MT544422 | CK/CH/AH/X0420/2019 | China | 2016 | GI-19 | KC577385 | 43SD-96III | China | 1996 | GI-19 |
| MN127993 | CK/CH/SD/0716/2016 | China | 2016 | GI-19 | KX252791 | ck/CH/LLN/98I | China | 1998 | GI-19 |
| MW877667 | IBV/Korea/2696 | Korea | 2019 | GI-19 | GQ253485 | IBV/La/SP/116/09 | Spain | 2009 | GI-19 |
| MN615461 | CK/CH/HuB/20180524 | China | 2016 | GI-19 | EF079116 | NL/L-1449T/04 | Netherlands | 2004 | GI-19 |
| MN615438 | CK/CH/HeN/20160330 | China | 2016 | GI-19 | MK581205 | gammaCoV/Ck/Poland/548/2004 | Poland | 2004 | GI-19 |
| MN128009 | CK/CH/LN/0927/2018 | China | 2016 | GI-19 | ON951677 | CK/CH/SX/MJ17 | China | 2016 | GI-19 |
| MW877612 | IBV/Korea/224 | Korea | 2019 | GI-19 | KY933090 | L1148 | United_Kingdom | 2017 | GI-19 |
| MW877613 | IBV/Korea/63 | Korea | 2020 | GI-19 | AY790363 | K10217-03 | Korea | 2003 | GI-19 |
| MW877632 | IBV/Korea/76 | Korea | 2019 | GI-19 | MT984595 | D1783/2/3/2011/RO | Romania | 2011 | GI-19 |
| MW877636 | IBV/Korea/151 | Korea | 2019 | GI-19 | DQ400359 | IS/1201 | Israel | 2004 | GI-19 |
| MN127995 | CK/CH/LN/0626/2017 | China | 2016 | GI-19 | FJ807923 | K283/04 | Korea | 2004 | GI-19 |
| MT544425 | CK/CH/LN/X0107/2019 | China | 2016 | GI-19 | OM525804 | D591/2/GR/05 | Greece | 2005 | GI-19 |
| MN128006 | CK/CH/AH/1207/2018 | China | 2016 | GI-19 | JQ991523 | RF/08/2010 | Russia | 2010 | GI-19 |
| MN615426 | CK/CH/HeN/20180304 | China | 2016 | GI-19 | FN430414 | ITA/90254/2005 | Italy | 2005 | GI-19 |
| MW877631 | IBV/Korea/61 | Korea | 2018 | GI-19 | AY790364 | K1255-03 | Korea | 2004 | GI-19 |
| MG734780 | CK/CH/GX/YL17-1 | China | 2016 | GI-19 | OM525805 | D683/HU/06 | Hungary | 2006 | GI-19 |
| MT162603 | I0812/19 | China | 2019 | GI-19 | KF297571 | KG3P | United_Kingdom | 2016 | GI-19 |
| MN614445 | IBV-SDDY-171225 | China | 2016 | GI-19 | MT984589 | D1617/3/2011/UA | Ukraine | 2011 | GI-19 |
| MH020185 | CK/CH/HD/171018 | China | 2016 | GI-19 | MK491747 | 2123 | Italy | 2017 | GI-19 |
| MF447723 | CK/CH/GX/NN16-4 | China | 2016 | GI-19 | MK491690 | 756 | Italy | 2014 | GI-19 |
| MT544420 | CK/CH/JL/U0525/2016 | China | 2016 | GI-19 | MK491734 | 18222 | Italy | 2016 | GI-19 |
| MN629010 | CK/CH/SX/BEI | China | 2016 | GI-19 | MK491673 | 285 | Italy | 2012 | GI-19 |
| MT544416 | CK/CH/HuB/X0329/2019 | China | 2016 | GI-19 | MK491678 | 14802 | Italy | 2013 | GI-19 |
| MN615425 | CK/CH/HeN/20180923 | China | 2016 | GI-19 | PP871397 | MR23/6 | Mongolia | 2023 | GI-19 |
| MN127987 | CK/CH/JS/1025/2016 | China | 2016 | GI-19 | JX840411 | YX10 | China | 2010 | GI-19 |
| MF447697 | CK/CH/JS/LYG17 | China | 2016 | GI-19 | OK507216 | CK/CH/MY/2020 | China | 2020 | GI-19 |
| MN055628 | HeN-2/China/2019 | China | 2016 | GI-19 | KY047602 | gammaCoV/Ck/Poland/G052/2016 | Poland | 2016 | GI-19 |

Supplementary Table 3. Pairwise comparison of nucleotide homology of coding sequences (CDS) between the Mongolian infectious bronchitis virus (MR23-6) and other IBV strains

| IBV strain ^a^ | GenBank accession no. | Nucleotide identity (%) ^b^ | | | | | | | | | | | | | |
| --- | --- | --- | --- | --- | --- | --- | --- | --- | --- | --- | --- | --- | --- | --- | --- |
|  |  | Complete genome | 1ab | 1a | 1b | S | 3a | 3b | E | M | 5a | 5b | N | 6b |  |
| Avian coronavirus strain D2002/2/2012/PT | MT984598 | 91.38 | 91.39 | 89.43 | 94.29 | 92.60 | 85.63 | 86.15 | 87.234 | 90.533 | 97.98 | 95.18 | 96.01 | 68 |  |
| Avian coronavirus strain D1760/2/2/2011/GR | MT984594 | 93.38 | 93.56 | 93.45 | 93.74 | 93.38 | 86.20 | 80.51 | 90.07 | 91.34 | 94.44 | 93.57 | 96.58 | 99.55 |  |
| Avian coronavirus strain D2220/2/2013/GR | MT984599 | 92.13 | 91.80 | 90.70 | 93.45 | 93.53 | 86.78 | 75.89 | 85.10 | 93.93 | 92.92 | 99.59 | 99.26 | 96 |  |
| Infectious bronchitis virus isolate D591/2/GR/05 | OM525804 | 92.65 | 92.72 | 92.94 | 92.42 | 93.32 | 84.48 | 76.41 | 85.81 | 90.82 | 98.48 | 93.97 | 96.82 | N.A.^c^ |  |
| Infectious bronchitis virus isolate CK/CH/MY/2020 | OK507216 | 92.66 | 94.01 | 95.11 | 92.41 | 88.39 | 86.20 | 75.38 | 85.19 | 87.13 | 89.39 | 91.96 | 95.77 | 100 |  |
| Infectious bronchitis virus strain CK/CH/SCMY/160315 | MT505388 | 92.15 | 93.86 | 94.81 | 92.52 | 87.79 | 85.63 | 75.89 | 87.14 | 90.99 | 88.38 | 93.17 | 93.90 | 57.56 |  |
| Infectious bronchitis virus strain ck/CH/SCYB/140913 | KU356856 | 91.83 | 94.11 | 94.66 | 93.35 | 87.21 | 85.63 | 75.38 | 85.56 | 87.27 | 89.39 | 92.36 | 88.37 | 58.82 |  |
| Infectious bronchitis virus isolate IBVUkr27-11 | KJ135013 | 92.16 | 93.75 | 93.90 | 93.56 | 81.96 | 86.20 | 82.56 | 88.65 | 94.67 | 98.48 | 94.37 | 94.39 | 100 |  |
| Infectious bronchitis virus isolate IBV/India/ck/03/23 | OR824987 | 91.86 | 93.31 | 93.66 | 92.83 | 81.64 | 86.78 | 86.15 | 87.23 | 92.16 | 92.42 | 100 | 99.91 | 100 |  |
| Infectious bronchitis virus isolate D722/SK/06 | OM525806 | 90.79 | 90.75 | 89.89 | 92.05 | 92.12 | 92.52 | 80 | 86.52 | 92.89 | 90.90 | 92.77 | 90.65 | N.A.^c^ |  |
| Infectious bronchitis virus strain gammaCoV/Ck/Poland/548/2004 | MK581205 | 89.98 | 89.43 | 89.25 | 89.75 | 94.28 | 86.78 | 87.17 | 86.17 | 93.49 | 95.45 | 93.17 | 90.32 | 73.33 |  |
| Infectious bronchitis virus isolate gammaCoV/Ck/Poland/G052/2016 | KY047602 | 91.66 | 92.96 | 92.32 | 93.93 | 82.83 | 86.20 | 81.02 | 86.87 | 94.84 | 97.98 | 94.77 | 93.17 | 99.11 |  |
| Infectious bronchitis virus strain Italy02 | MN548288 | 89.66 | 91.27 | 89.47 | 93.96 | 81.23 | 85.05 | 81.02 | 90.07 | 93.52 | 96.97 | 93.97 | 91.54 | 56.41 |  |
| Infectious bronchitis virus strain gammaCoV/Ck/Poland/80/1989 | MK581202 | 88.44 | 89.77 | 88.40 | 91.82 | 80.45 | 86.20 | 86.66 | 89.47 | 88.60 | 89.89 | 94.77 | 90.65 | 78.50 |  |
| Infectious bronchitis virus strain gammaCoV/Ck/Poland/162/1997 | MK581203 | 89.28 | 90.64 | 89.12 | 92.93 | 80.10 | 92.52 | 81.53 | 88.84 | 95.14 | 95.96 | 95.58 | 91.46 | 72.88 |  |
| Infectious bronchitis virus strain gammaCoV/Ck/Poland/255/1997 | MK581204 | 89.25 | 90.56 | 89.46 | 92.24 | 81.78 | 86.78 | 79.48 | 88.48 | 91.86 | 96.46 | 94.37 | 90.56 | 72 |  |
| Infectious bronchitis virus isolate ck/CH/LJX/2017/07 | MN307884 | 86.97 | 86.51 | 84.63 | 89.36 | 86.41 | 83.90 | 76.41 | 85.40 | 89.49 | 84.34 | 91.56 | 96.91 | 100 |  |
| Infectious bronchitis virus strain ck/CH/LHLJ/08-6 | KX252788 | 87.6 | 86.48 | 84.58 | 89.34 | 96.95 | 83.33 | 76.92 | 86.47 | 89.05 | 84.84 | 91.96 | 87.39 | 68 |  |
| Infectious bronchitis virus strain ck/CH/LSD/101223 | KX364299 | 87.86 | 86.75 | 84.48 | 90.17 | 96.34 | 82.75 | 73.84 | 86.47 | 89.20 | 92.92 | 100 | 88.04 | 72.44 |  |
| Infectious bronchitis virus strain YX10 | JX840411 | 87.68 | 86.58 | 84.73 | 89.39 | 96.86 | 83.33 | 76.41 | 86.47 | 89.34 | 85.85 | 93.57 | 86.58 | 72.44 |  |
| Turkey coronavirus strain gammaCoV/Tk/Poland/G160/2016 | MT367412 | 84.91 | 91.17 | 90.21 | 92.63 | 47.70 | 91.37 | 88.71 | 89.71 | 89.80 | 98.48 | 93.17 | 92.29 | 71.22 |  |

^a^ All sequence data were retrieved from the GenBank database.

^b^ Nucleotide identity percentages were calculated using pairwise sequence alignment using MAFFT v7.308. Highest identity for each coding sequence was underlined.

^c^ N.A. = Not available.

Supplementary Table 4. Recombination events detected in whole genome of the Mongolian infectious bronchitis virus isolate (MR23/6)

| Major parental strain^a^ | Minor parental strain^a^ | p-values of the detection methods^b^ | | | | | | |
| --- | --- | --- | --- | --- | --- | --- | --- | --- |
|  |  | RDP | GENECONV | MaxChi | Chimera | 3Seq | Bootscan | SiScan |
| IBVUkr27-11  (KJ135013) | YX10  (JX840411) | 4.635×10^-157^ | 9.655×10^-186^ | 4.620×10^-197^ | 4.620×10^-197^ | 1.606×10^-212^ | 3.511×10^-199^ | 7.821×10^-54^ |
| ck/CH/LJX/2017/07  (MN307884) | CK/CH/MY/2020  (OK507216) | 2.338×10^-30^ | 3.487×10^-35^ | 4.620×10^-197^ | 2.200×10^-16^ | 3.350×10^-7^ | 1.376×10^-29^ | 4.914×10^-70^ |
| D2220/2/2013/GR  (MT984599) | CK/CH/MY/2020  (OK507216) | 7.238×10^-24^ | 1.732×10^-19^ | 8.141×10^-14^ | 5.371×10^-14^ | 1.862×10^-9^ | 7.440×10^-23^ | 2.080×10^-16^ |
| CK/CH/SCMY/160315 (MT505388) | gammaCoV/Ck/Poland/548/2004 (MK581205) | 2.034×10^-10^ | 1.082×10^-7^ | 3.905×10^-13^ | 9.143×10^-12^ | 1.709×10^-13^ | 7.998×10^-11^ | 8.892×10^-15^ |
| D2220/2/2013/GR (MT984599) | gammaCoV/Ck/Poland/G052/2016 (KY047602) | 2.699×10^-5^ | 2.515×10^-7^ | 9.863×10^-7^ | 1.057×10^-3^ | 1.787×10^-7^ | N.S.^c^ | N.S.^c^ |

^a^ The major and minor parental strain is the virus contributing the larger fraction of the recombinant sequences and the smaller fraction of the recombinant sequences to the generation of recombinant IBV, respectively. Strain information includes a detailed description of each strain, followed by the corresponding DNA accession number in parentheses for reference.

^b^ Recombination events were confirmed when P-values were < 1 × 10^-2^ from at least five detection methods.

^c^ N.S. = Not Significant
