# Supplementary figures and images for: First report and complete genome analysis of infectious bronchitis virus from retailed chicken meat in Mongolia in 2023
Source: Front Vet Sci. 2024 Dec 13;11:1465342. doi: 10.3389/fvets.2024.1465342 (PMC11671396; doi:10.3389/fvets.2024.1465342)

## Slide 1
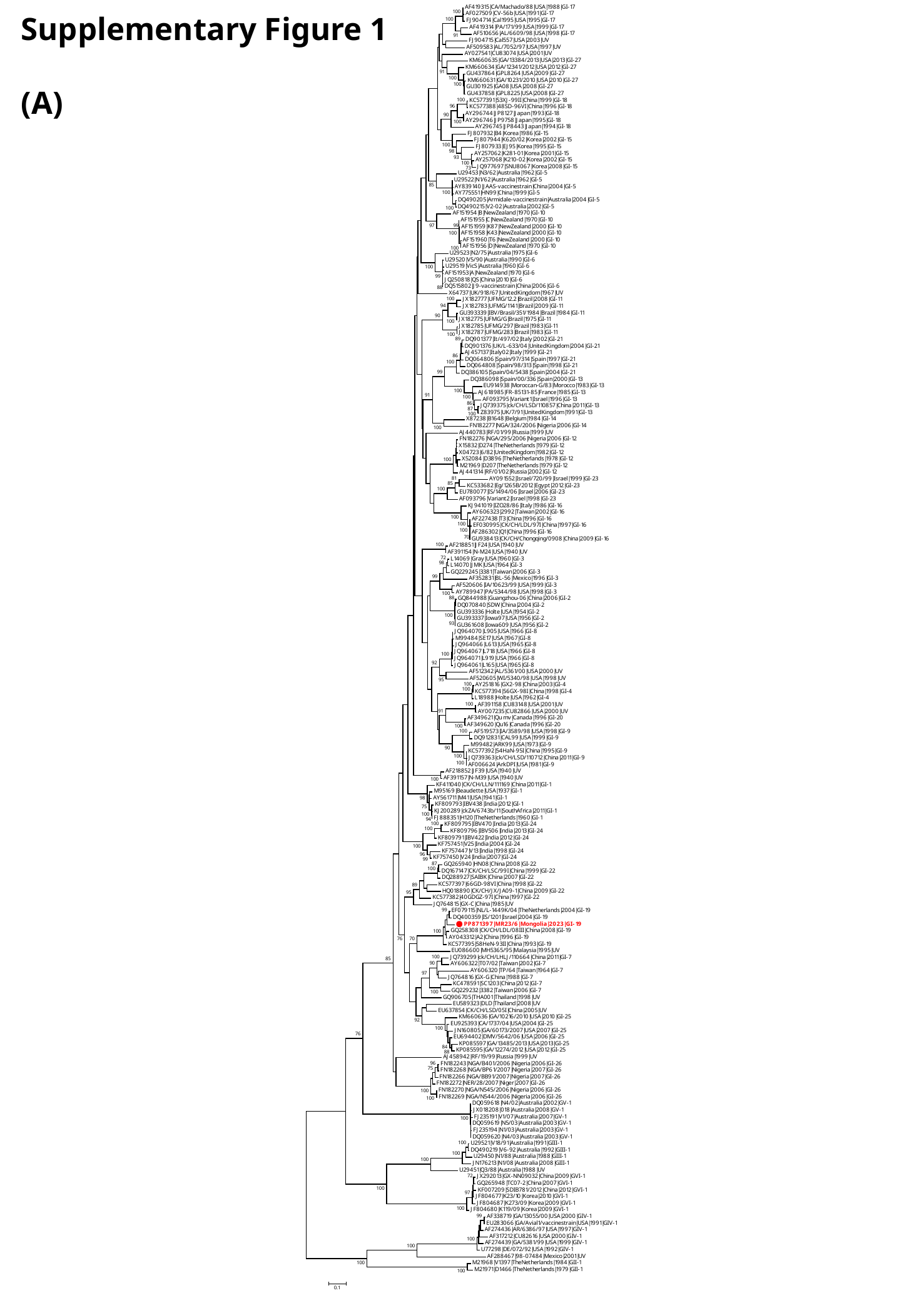

Supplementary Figure 1
(A)

## Slide 2
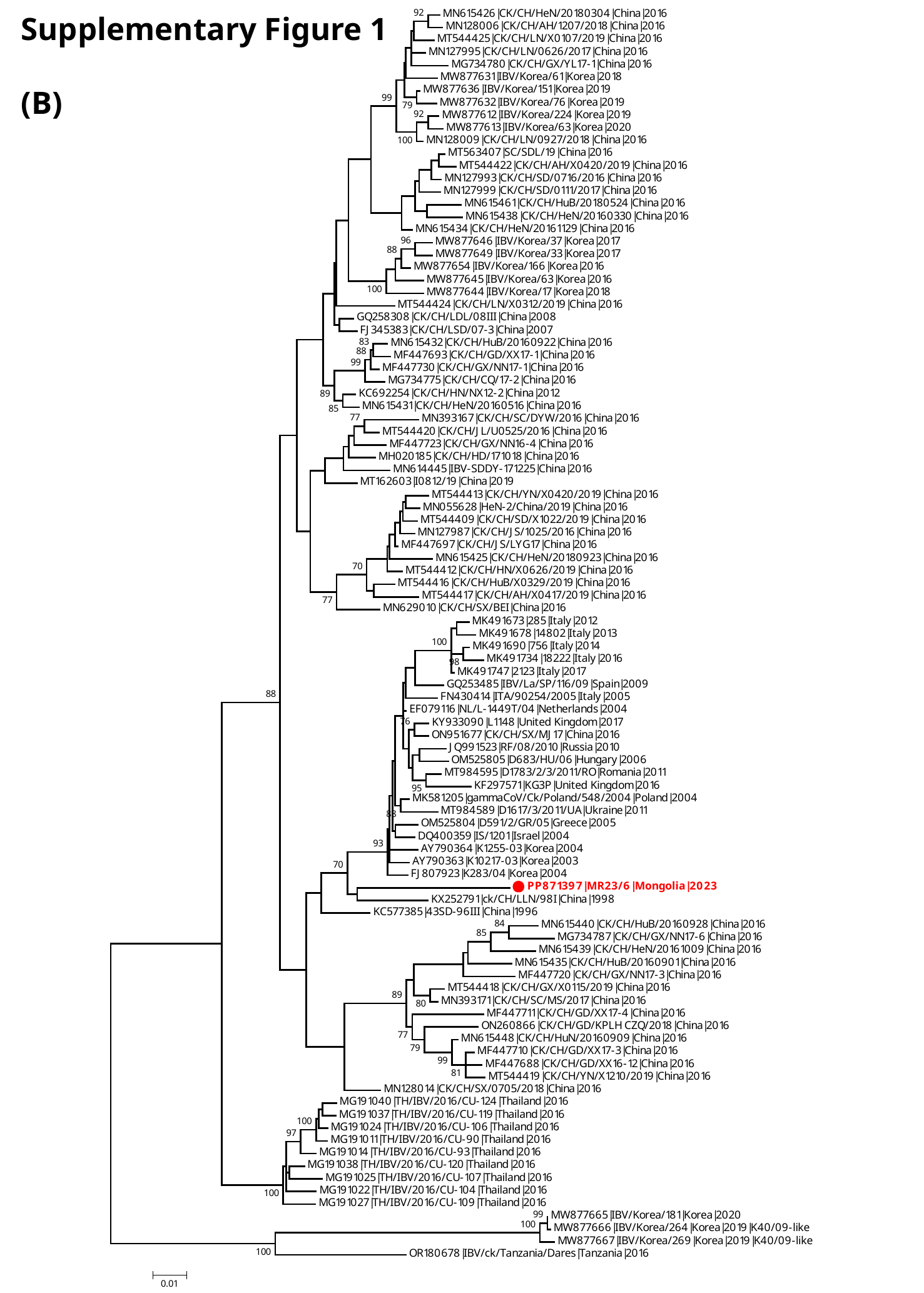

Supplementary Figure 1
(B)

Supplement: Supplementary Figure 1 — (A) Phylogenetic trees based on alignment of S1 gene sequences of the Mongolian infectious bronchitis virus (IBV) isolate (red) and reference strains. The trees were created by the maximum-likelihood method in RAxML using Tamura-Nei model and 1,000 bootstrap replicates. The IBVs newly isolated in this study are shown in red with a circle. S1 gene-based phylogenetic tree was constructed using 191 reference sequences. (B) Phylogenetic trees based on alignment of S1 gene sequences of the Mongolian infectious bronchitis virus (IBV) isolate (red) and reference strains. The trees were created by the maximum-likelihood method in RAxML using Tamura-Nei model and 1,000 bootstrap replicates. The IBVs newly isolated in this study are shown in red with a circle. S1 gene-based phylogenetic tree was constructed using 101 IBV strains previously reported as GI-19 lineage. [file Presentation_1.pptx]
